# The impact of Internet-based healthcare derived from the COVID-19 pandemic on outpatients in a cardiology department

**DOI:** 10.3389/fdgth.2025.1475422

**Published:** 2025-02-18

**Authors:** Jie Liu, Yunhan Fei, Ying Gao, Yu Meng, Dongxue Huang, Wenjuan Zhao, Keliang Xie

**Affiliations:** ^1^ Department of Critical Care Medicine, Tianjin Medical University General Hospital, Tianjin, China; ^2^ Department of Emergency, Tianjin Huanhu Hospital, Tianjin, China; ^3^ Department of Nurology, Tianjin Medical University General Hospital, Tianjin, China

**Keywords:** Internet-based online medical service, cardiology department, grade A hospital, medical quality, medical model

## Abstract

**Objective:**

This study examines the impact of a COVID-19 pandemic-derived online medical service on cardiovascular patient visits and assesses whether these services can ease the strain on medical resources.

**Method:**

This study investigated the impact of the COVID-19 pandemic on cardiology services and hospital operations. We analyzed key primary medical indicators in cardiology, including outpatient visits, inpatient improvement rates, cure rates, and mortality rates, over three years from 2019 to 2021. Furthermore, the study assessed the influence of the development of Internet-based medical services on the treatment of cardiovascular disease. Specifically, we compared the changes in the number of outpatient visits in four categories of offline outpatient clinics in the Department of Cardiology during two phases: Phase I (1 February 2019 to 28 February 2020) and Phase II (1 March 2020 to 28 February 2021).

**Results:**

Compared to the period before online services (T1), the second stage (T2) saw a significant decrease in total offline and general clinic visits. After the establishment of the online clinic, the third period (P3) showed a significant reduction in total offline, general, and senior clinic visits compared to the first period (P1), while vice-senior and VIP/international clinic visits increased. The number of online clinic visits and VIP/international clinic visits continued to rise. Online consultations had the highest proportion (55.9%), while prescriptions and examinations had the lowest (3.3%), although they showed a gradually increasing trend. After the implementation of the online clinic, the improvement rate of patients' conditions increased and the mortality rate decreased.

**Conclusion:**

Since the advent of online medical services, cardiovascular patients have increasingly opted for online diagnosis and treatment. Since March 2021, the online outpatient service has driven the overall growth in hospital outpatient numbers while maintaining medical quality. The primary use of the online medical service is for consultations, which shortens medical time and reduces implicit costs for patients.

## 1 Introduction

Since late 2019, infections with the SARS-CoV-2 virus have caused the COVID-19 pandemic, which is a major challenge to global health (1). The lack of specific treatments and vaccines has forced managers to focus on prevention strategies. To control the spread of the virus, the WHO has implemented various public health policies, including the use of masks, correct and frequent hand washing, social distancing, and isolation strategies (2). This has been an unprecedented challenge for the health sector, forcing it to change the way services are commonly delivered (3–5). Through this driving force, health agencies have moved care from the hospital to the patient's home by strengthening home care and implementing telemedicine, which is a frontline tool in tackling the disease (6).

Internet medical services are considered an ideal tool to deal with this kind of emergency. Internet telemedicine has been gradually implemented worldwide for approximately 10 years. However, the slow development of Internet medical services is due to the lack of relevant laws and regulations, the limited economic investment of hospitals in technical resources, and the reluctance of some medical providers and patients to adopt telemedicine (6–8). The COVID-19 pandemic has promoted and accelerated the development of Internet medical services (9).

However, how the opening of Internet-based online clinics and other medical services will affect the work of offline outpatients in the hospital and how to adjust the medical service work in the main body of the hospital according to these changes are questions that still need to be solved urgently. Therefore, this study collected the number of online and offline outpatients in the Cardiology Department of Tianjin Medical University General Hospital from March 2019 to August 2019 to study the impact of online medical services on the number of offline outpatients in the Cardiology Department of Tianjin Medical University General Hospital. This study aimed to clarify the number of patients using Internet-based outpatient clinics and their behavior and to investigate the impact of the opening of Internet-based online clinics on the number of patients using different types of offline outpatient clinics.

## 2 Methodology

### 2.1 Data collection

This study analyzed the number of offline clinic patients in the Department of Cardiology of Tianjin Medical University General Hospital from 1 February 2019 to 28 February 2021, and the number of online clinic patients in the Department of Cardiology of Tianjin Medical University General Hospital from 1 March 2020 to 31 August 2021. Taking the opening date of the online medical service (1 March 2020) as the node, the offline outpatient medical data were divided into two stages: stage 1 (T1, 1 February 2019 to 29 February 2020) and stage 2 (T2, 1 March 2020 to 28 February 2021). Furthermore, the period of March to August in 2019, 2020, and 2021 was divided into three periods: the first period (P1, 1 March 2019 to 31 August 2019) before the opening of the Internet-based outpatient service, and the second (P2, 1 March 2020 to 31 August 2020) and third (P3, 1 March 2021 to 31 August 2021) periods after the opening of the Internet-based outpatient service.

### 2.2 Definitions

In this study, the total number of patients in offline clinics, i.e., a VIP and international clinic, a senior clinic, a vice-senior clinic, and a general clinic, and in an online clinic, in different stages, periods, and groups were counted separately, and the mean, standard deviation, and trend of change in the number of patients in different types of clinics per month were calculated.

An offline clinic refers to a medical institution that patients personally visit for face-to-face diagnosis and treatment activities. An online clinic service is a telemedicine service through the Internet. In this study, “outpatient” refers to both online and offline outpatient services. A VIP and international clinic primarily caters to VIP clients, foreign nationals, and those with high-end healthcare needs. This clinic offers premium medical services, often including multilingual support, provides a more comfortable environment, and features streamlined appointment and consultation processes. The fees are relatively high, and some services may not be covered by social health insurance. A senior clinic is staffed by senior-level specialists, typically chief physicians, who possess extensive clinical experience. Appointments are often required in advance, and fees are higher than those for general outpatient services. This clinic is suitable for patients with complex or rare conditions that require expert consultation. A vice-senior clinic is led by physicians with vice-senior titles, such as associate chief physicians, who have considerable professional experience and expertise. This service is appropriate for patients with relatively complex conditions that may not necessitate a senior-level specialist. Fees are moderately higher than general outpatient rates. A general clinic provides basic outpatient services for the majority of patients, typically managed by junior or mid-level physicians. The fees are lower, and coverage by social health insurance is generally higher. This clinic is for common illnesses and basic medical needs.

Moreover, in this study, internet medical behavior includes medical consultation, medical examination, prescription, and examination + prescription. In a medical consultation, patients inform doctors of their health status, and doctors make preliminary diagnoses of possible diseases based on the patient's complaints and medical history. Regarding examinations, some patients need to undergo regular relevant laboratory tests. After the patient registers, the doctor conducts an examination and laboratory tests based on the preliminary diagnosis to further clarify the patient's current health condition. Laboratory tests may include blood tests, urine tests, and imaging examinations (such as X-rays, CT, and MRI). Prescription refers to requesting prescription drugs from doctors, and doctors prescribing drugs after fully diagnosing the patient's condition.

### 2.3 Internet-based healthcare

The Internet-based clinic used in this study is called Careate Q Medicine (Qmed) [Careate Medical Technology (Tianjin) Co., Ltd] (10). It is designed to enhance patient experience and streamline hospital management by providing a versatile platform with a clean, intuitive user interface (UI) for patients, healthcare providers, and administrators. Each interface is optimized for ease of navigation, offering user-friendly functions and efficient workflows (Supplementary Figure S1).

The core functionalities of Qmed include the following:

• Appointment scheduling: Patients can book appointments with their preferred departments and doctors online, reducing wait times and simplifying the scheduling process.

• Electronic health records (EHRs): Patients can access their medical history, test results, and medication records, ensuring continuity of information and enabling better personal health management.

• Telemedicine: The app supports real-time communication between patients and doctors through text, voice, and video, enabling consultations for minor issues or follow-up appointments without needing a physical visit.

• Payment and insurance claims: Users can make payments for registration and consultation fees directly within the app, with support for various payment methods and, in some versions, insurance claims processing.

• In-hospital navigation: Qmed provides a navigation tool to help patients locate departments, laboratories, and pharmacies within the hospital, saving time and reducing confusion.

Furthermore, usage overview includes:

• User registration and login: Patients and doctors register using their phone numbers, creating a secure login process. Patients can input basic health information, while doctors provide credential verification.

• Appointment and payment: Patients can select their department, doctor, and preferred time slot, with options to pay digitally, ensuring a seamless booking and payment experience.

• Health records and telemedicine: Patients can view their health records and engage in telemedicine consultations. Doctors use their interface to review patient histories, schedule consultations, and issue prescriptions. Tianjin Medical University General Hospital has established a strict data management system in collaboration with Careate Medical Technology Co., Ltd., encrypting and storing user data, and regularly conducting data backups and security checks. Furthermore, the hospital has collaborated with legal institutions to establish legal data protection barriers.

### 2.4 Statistical analysis

SPSS 25.0 and GraphPad Prism 9.2.0 were used for the statistical analyses to compare the monthly number of cardiovascular outpatients in multiple stages and periods, including the total number using offline and online clinics, and those using a VIP and international clinic, senior clinic, vice-senior clinic, and general clinic. The monthly number of patients is represented as the mean ± standard deviation. A *t*-test was performed for comparisons between groups and *P* < 0.05 was considered statistically significant. We calculated the monthly proportion of different medical behaviors among the outpatients in the Internet-based online clinic in multiple groups and performed a *t*-test between each group, with *P* < 0.05 considered statistically significant. Using Joinpoint software, a trend analysis of the number of outpatients in various categories in multiple quarters was conducted, and *P* < 0.05 was considered statistically significant.

Joinpoint was also used for trend analysis to calculate the changing trend of different medical data. A Joinpoint regression model was set up as a log-linear model (ln *y* = *xb*) and an unrelated model was chosen for fitting. The grid search modeling method was applied in this study. *P* < 0.05 was considered statistically significant. Joinpoint 4.9.0.0 was used for the statistical analysis (11).

## 3 Results

### 3.1 Changes in the number of outpatient visits due to the Internet-based online clinic

**Figure 1:**
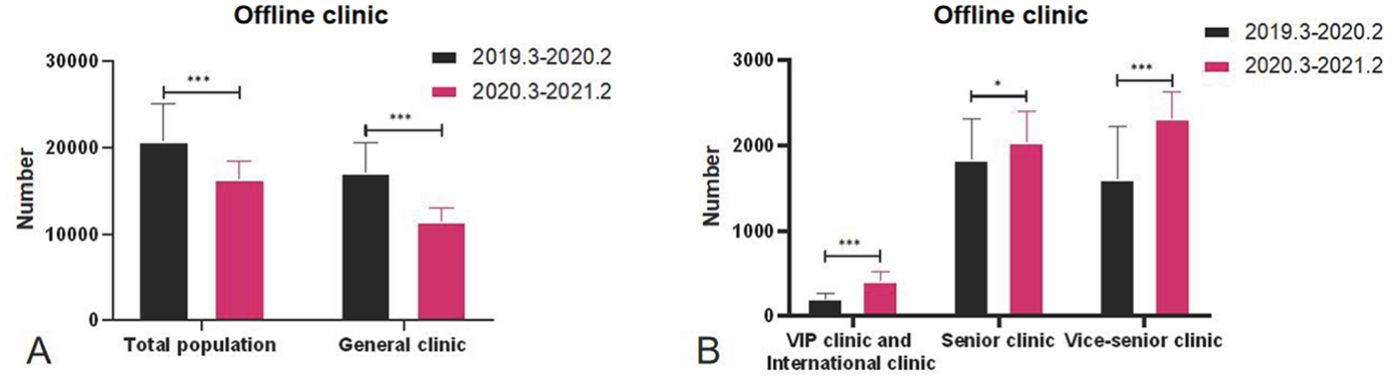
**(A)** Comparison of the overall number of outpatient clinics. **(B)** Comparison of the number of visits to different types of outpatient clinics.

**Figure 2:**
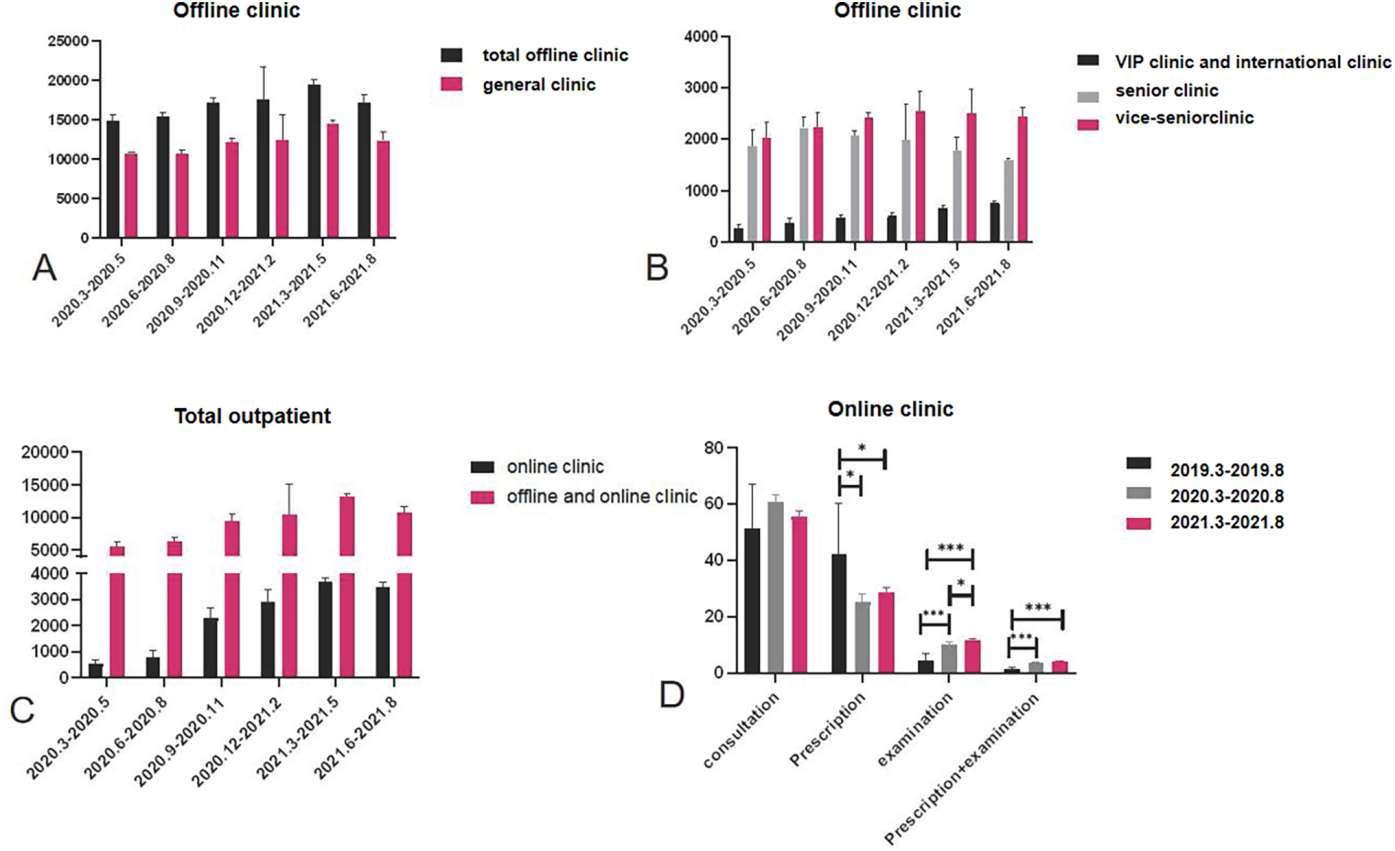
**(A–C)** Comparison of the number of outpatients in different clinics in different quarters. **(D)** With the development of online clinics, hospitalization behavior has changed. The proportion of prescriptions decreased gradually, while that of examinations increased.

Compared with T1, i.e., after the Internet medical treatment service was opened, the number of outpatient visits to the VIP and international clinic, senior clinic, and vice-senior clinic increased significantly in T2. Specifically, the number of outpatient visits to the VIP and international clinic and the vice-senior clinic very significantly increased (*P* < 0.001), while the total number of outpatient visits to the offline clinic and the general clinic decreased significantly (Table 1, Figure 1). Since the establishment of Internet-based medical services, the number of outpatient visits to the online clinic gradually increased, with a very significant statistical difference (Table 2, Figure 2).

**Table 1:** Changes in the number of offline outpatient visits to the Cardiology Department in the hospital (*x* ± *s*).

	1 February 2019–29 February 2020	1 March 2020–28 February 2021	*P*
VIP clinic	201.9 ± 65.7	412.3 ± 113.8	<0.001
Senior clinic	1,824.2 ± 481.5	2,036.8 ± 366.9	0.042
Vice-senior clinic	1,600.9 ± 615.6	2,307.7 ± 317.5	<0.001
General clinic	17,057.8 ± 3498.0	11,505.6 ± 1,638.1	<0.001

**Table 2:** Changes in the number of online outpatient visits to the Cardiology Department in the hospital (*x* ± *s*).

Time period	Outpatient volume (*x* ± *s*)	*P*
March 2020 to May 2020	560.3 ± 125.9	—
June 2020 to August 2020	810 ± 231.0	0.038
September 2020 to November 2020	2,287.7 ± 397.2	<0.001
December 2020 to February 2021	2,916.7 ± 482.6	0.170

Compared with P1, after the opening of the Internet online clinic, the total number of outpatient visits to the offline clinics and general clinic decreased significantly (*P* < 0.001) in P2, while the number of outpatient visits to the VIP and international clinic (*P* < 0.05) and vice-senior clinic (*P* < 0.001) increased significantly. Compared with P1, the total number of outpatient visits to the offline clinics (*P* < 0.001), general clinic (*P* < 0.001), and senior clinic (*P* < 0.05) was significantly reduced in P3, while the number of outpatient visits to the VIP and international clinic and vice-senior clinic (*P* < 0.001) increased significantly. Compared with P2, there was a significant increase in the total number of outpatient visits to the offline clinics, general clinic, and VIP and international clinic (*P* < 0.001) in P3 and a significant decrease in the number of outpatient visits to the senior clinic (*P* < 0.05) (Figure 2).

According to the analysis of the quarterly number of patients, since the establishment of the Internet-based medical service, the number of outpatient visits to the Internet-based online clinic (*P* < 0.01), vice-senior clinic (*P* < 0.05), VIP and international clinic (*P* < 0.001), and offline and online clinics (*P* < 0.05) showed an increasing trend. The number of outpatient visits to the online clinic, and VIP and international clinic continued to increase, while the number of outpatient visits to the senior clinic decreased (Figure 2).

### 3.2 Analysis of medical behavior in the Internet-based online clinic

In this study, the proportion of consultations in the Internet-based online clinic was the highest (55.8%) without a significant difference (*P* > 0.05), and the proportion of prescription + examination was the lowest (3.3%). The proportion of prescriptions through the Internet-based online clinic significantly decreased between P1 and P3 (42.42%; 25.39; 28.57%, *P* < 0.05). Furthermore, the proportion of examinations (4.68%; 10.11%; 11.86%, *P* < 0.05) and prescription + examination (1.59%; 3.65%; 3.95%, *P* < 0.05) through the Internet-based online clinic gradually increased between P1 and P3.

### 3.3 Healthcare safety and quality

**Figure 3:**
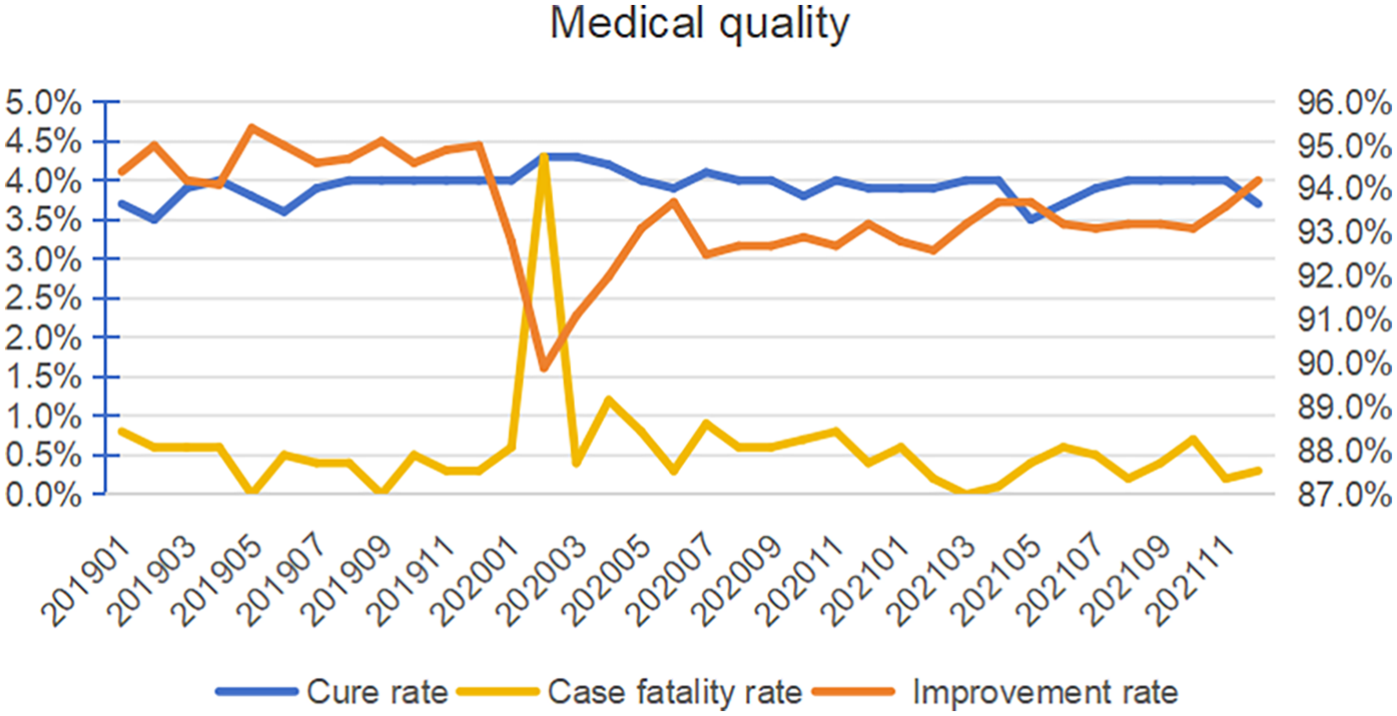
Trend analysis of medical service capability. The right vertical axis represents the patient improvement rate.

Although Internet-based medicine significantly increased the overall number of patients with cardiovascular diseases, it has not affected the medical safety and quality of the hospital. In this study, the improvement rate of hospitals has significantly increased after the implementation of Internet medicine, with a monthly change percentage of 1% (95% CI: 0,0.1; *P* = 0.013). The mortality rate has no significant change (*P* = 0.626) (Table 3, Figure 3).

**Table 3:** Medical performance capability trends.

Characteristic	Joinpoint	MPC	95% CI	*P*
Improvement rate	S1	−0.03	−0.1 to 0	0.001
	S2	0.01	0 to 0.1	0.001
Case fatality rate	—	0.38	−1.2 to 2.0	0.626
Cure rate	—	0	—	0.809

S1, first stage; S2, second stage; MPC, monthly percentage change.

## 4 Discussion

Before the COVID-19 pandemic, China's Internet-based medical platforms were mainly provided by companies providing online services, and doctors of all levels from all over the country used their medical platforms. Since the COVID-19 pandemic, to ensure the delivery of medical services to control the spread of the pandemic, medical institutions at all levels quickly built their own Internet-based medical platforms, especially tertiary hospitals, which are used to independently carry out various online consultations and provide online and offline medical services. These not only involve online consultations and inquiries but also involve online prescriptions, appointments for examinations, online readings, drug delivery, and patient follow-ups. Thus, hospitals have used media and communication technologies to provide comprehensive digital medical services and better serve the health needs of their patients.

Since March 2020, many hospitals in the United States have experienced a massive migration to virtual care, resulting in a reduction in in-person patient visits of more than 80%. At the height of the pandemic, a report in New York City indicated that the number of Internet consultations had increased from less than 50 per day to more than 1,000 per day, accounting for more than 70% of hospital outpatients (12). Furthermore, in Italy, the United States, and India, the percentage of migration from offline medical care to online medical care is 60%–95% (13–15). However, international studies on Internet medical care mainly focus on the technical aspects, such as the combination of telemedicine and mobile medical care, but Internet-based hospitals, which are a new medical service mode, are rarely studied at present. Because China's medical system is different from that of developed countries, it is difficult for researchers to determine the impact of the establishment of Internet-based hospitals on offline medical services using the model of telemedicine from developed countries (16, 17). Internet-based healthcare in the European Union, the United States, India, and other countries focuses on the development of telemedicine and electronic medical records in mobile medicine, providing telemedicine service guidance and rehabilitation suggestions for cardiovascular, dermatology, and stroke patients (18–20). This rests on the premise of promoting patients’ health by collecting patient medical treatment data, including all kinds of test results, and medical information, and analyzing these data. However, with the repeated fluctuations of the pandemic, what we urgently need to solve is how not to affect patients' medical treatment while reducing crowding. Therefore, the General Hospital of Tianjin Medical University has established an Internet-based outpatient platform based on national conditions. However, the influence of an Internet-based outpatient service on patients' medical behavior and whether it meets patients' medical needs at present are still unclear.

According to the results of this study, the opening of the online outpatient clinic has met the medical needs of the majority of patients during the pandemic, and the number of online doctors has increased significantly from quarter to quarter. Furthermore, the total number of outpatient visits to offline clinics and the general clinic was significantly reduced. Most patients were more likely to use the Internet-based outpatient medical form. Furthermore, simple outpatient behaviors using Internet-based medical services, such as prescribing medicine and making appointments for examinations, gradually increased. The results show that Internet-based medical treatment not only facilitated the patients' medical treatment but also reduced the burden of medical treatment and the service cost of the medical institution.

After the introduction of Internet medical services, the number of visits to the offline general and senior clinics significantly dropped, while there was an increase in the number of outpatient visits to the VIP and international clinic. This shift may imply a redistribution of patient volume, affecting traditional outpatient services. The research results provide a theoretical foundation and basis for tertiary medical institutions to better implement Internet-based medical care. However, some patients, particularly older adults, may struggle to adapt to online services due to a lack of digital literacy, which could impact their access to care. We emphasize the importance of multi-channel communication methods to ensure that these patients receive the necessary support via telephone or in-person guidance, thereby improving their online healthcare experience and ensuring equitable access to medical services.

There are also some limitations in this study. First, it is a single-center study, which cannot represent the nationwide data. Second, the specific diseases diagnosed are not analyzed in this study, so the results of this study need to be supplemented by further research. Third, the research institution is located in northern China, where distinctly varied seasons contribute to monthly environmental fluctuations (e.g., temperature, humidity, and PM2.5 levels) that may influence cardiology outpatient visits. To minimize potential bias from seasonal changes, a horizontal comparison was conducted across the same seasonal time periods in different years to reduce the environmental impact on cardiology patient visits. Fourth, in adherence to user confidentiality principles, the research team has not yet extracted patient age data, and thus, an age-stratified analysis has not been performed. Further in-depth research, including age stratification, will be pursued following the refinement of relevant ethical protocols and regulatory approvals.

In addition, with the growth in demand for Internet-based medical services, it is particularly important to maintain a high level of diagnostic and treatment quality. In this study, the improvement rate of hospitals has significantly increased after the implementation of Internet medicine, and the mortality rate has no significant change. However, compared to traditional offline outpatient services, Internet-based outpatient care has certain limitations. For instance, physicians are unable to perform direct physical examinations or observe patients' behavioral changes, making it challenging to monitor and ensure the quality of online consultations and treatments.

## 5 Conclusion

Since the establishment of online medical services, patients are more and more inclined to choose online clinics. Since March 2021, Internet-based outpatient services have contributed to an overall increase in offline clinics in hospitals, with increasing numbers of patients willing to choose expert outpatient services, especially VIP and international clinics. Internet-based medical services not only meet the needs of patients for medical consultations but also reduce the waiting time and cost for patients. We suggest promoting online diagnosis and treatment services more widely.

## Data Availability

The raw data supporting the conclusions of this article will be made available by the authors, without undue reservation.
