# Supplementary figures and images for: The impact of Internet-based healthcare derived from the COVID-19 pandemic on outpatients in a cardiology department
Source: Front Digit Health. 2025 Feb 18;7:1475422. doi: 10.3389/fdgth.2025.1475422 (PMC11876138; doi:10.3389/fdgth.2025.1475422)

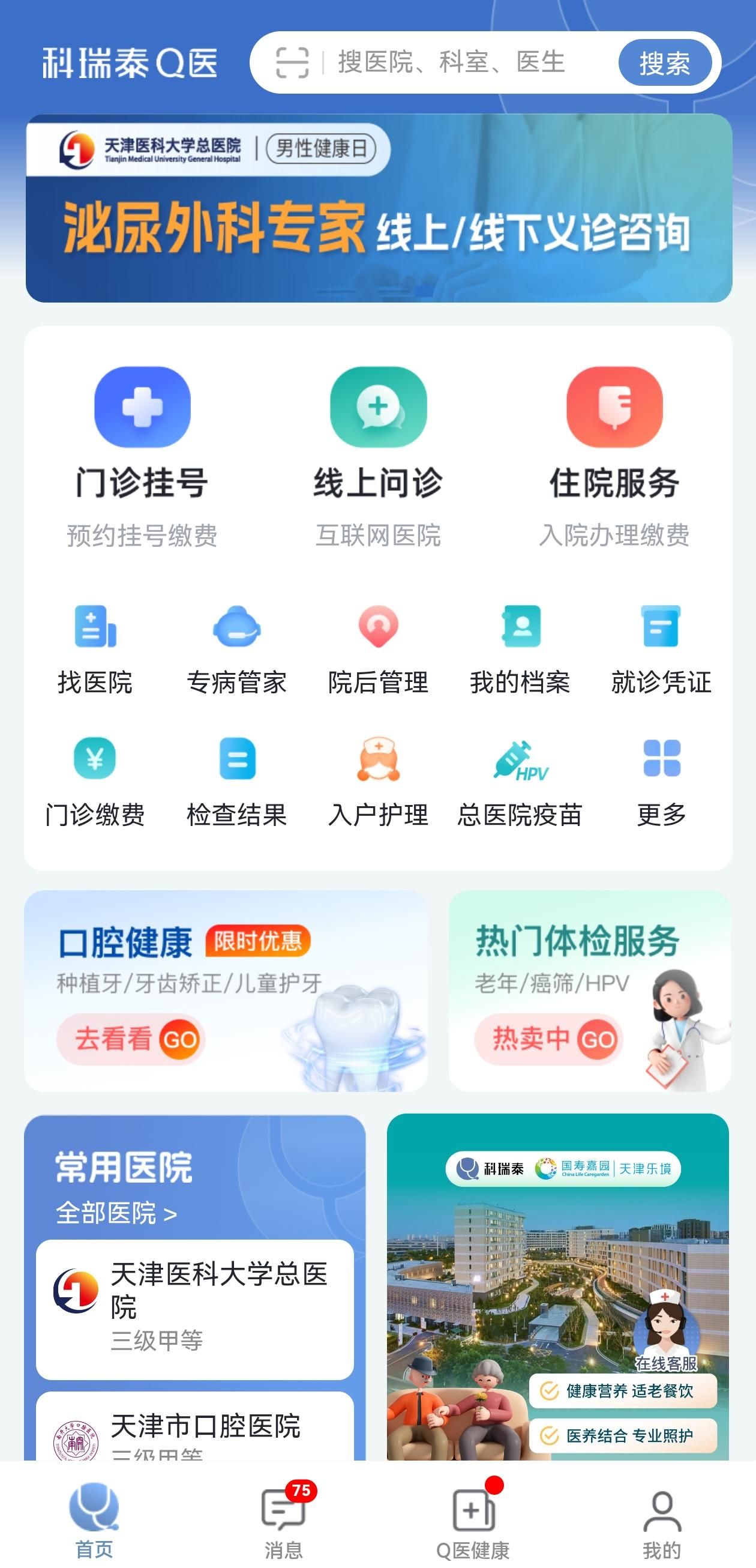

Supplement: Supplementary file 1 [file Image1.tif]
